# Inflammation at the Maternal–Fetal Interface: Mechanisms Linking Maternal–Fetal Immunity to Preeclampsia and Fetal Growth Restriction

**DOI:** 10.3390/ijms27093954

**Published:** 2026-04-29

**Authors:** Jezid Miranda, Natalia Maestre, Mariana Devia, Roberto Zapata, Margarita M. Ochoa-Díaz, Walter Annicchiarico

**Affiliations:** 1 Department of Obstetrics and Gynecology, Faculty of Medicine, University of Cartagena, Zaragocilla Carrera 50 #24-63, Cartagena de Indias 130014, Colombia; nmaestreromero@gmail.com (N.M.); robertorazs@hotmail.com (R.Z.); wanichiarico@gmail.com (W.A.); 2 Department of Obstetrics and Gynecology, Centro Hospitalario Serena del Mar, Cartagena de Indias 130008, Colombia; 3 Department of Obstetrics and Gynecology, University of Sinu, Cartagena de Indias 130015, Colombia; marianadeviacabrera@gmail.com; 4 Department of Obstetrics and Gynecology, Clinica General Del Norte, Barranquilla 080002, Colombia; 5 School of Medicine, Grupo de Investigación Básicas y Clínicas Facultad de Ciencias de Salud (GIBACUS), University of Sinu, Cartagena de Indias 130015, Colombia; mdochoadiaz@gmail.com

**Keywords:** preeclampsia, fetal growth restriction, placenta, maternal–fetal interface, inflammation, pregnancy complications, targeted therapy

## Abstract

Inflammation is a physiological and tightly regulated component of normal pregnancy, contributing to implantation, placental development, and the initiation of parturition. The placenta functions as an active immunological hub, coordinating innate and adaptive immune responses to maintain tolerance while protecting against infection. Preeclampsia and fetal growth restriction (FGR) are major causes of maternal and perinatal morbidity worldwide and represent central manifestations of placental disease. Increasing evidence indicates that these conditions share key pathophysiological mechanisms, including placental dysfunction and maladaptive maternal immune responses. When immune regulation at the maternal–fetal interface becomes disrupted, inflammatory pathways contribute to impaired placental development and vascular maladaptation. In this context, excessive immune activation—driven by inflammasome signaling, Th1/Th17 polarization, and altered natural killer and macrophage function—can compromise placental perfusion, promote antiangiogenic imbalance, and lead to systemic endothelial dysfunction. This review, therefore, focuses on how immune dysregulation contributes to placental dysfunction in preeclampsia and FGR, synthesizing current knowledge of the maternal–fetal immune interface and exploring therapeutic strategies that link pathogenic mechanisms to targeted interventions. A deeper understanding of placental immunology and inflammatory signaling is essential to develop precision therapies. Established therapies, including low-dose aspirin, low-molecular-weight heparin, and antenatal corticosteroids, aim to mitigate inflammation and optimize fetal outcomes, while adjunctive strategies target oxidative stress, nutritional deficits, and the maternal microbiome. Emerging approaches such as cytokine-targeted biologics, inflammasome inhibitors, and mesenchymal stem cell therapies show promise but require rigorous safety and efficacy evaluation. Future research should prioritize biomarker validation, pathway-specific interventions, and equitable implementation to reduce inflammation-driven pregnancy complications.

## 1. Introduction

Inflammation is a fundamental physiological process in pregnancy, supporting implantation, placental development, and the onset of labor [1,2,3,4]. Through finely regulated immune modulation, the maternal immune system maintains a delicate balance, tolerating the semi-allogeneic fetus while maintaining host defense. Successful pregnancy depends on tightly controlled temporal shifts in immune activity. A proinflammatory environment during early gestation facilitates implantation and trophoblast invasion, whereas a predominantly anti-inflammatory state during mid-gestation promotes placental maturation and fetal growth. Towards term, the re-emergence of inflammatory signaling contributes to the physiological onset of labor [4,5].

Increasing evidence suggests that placental inflammation is not merely a secondary consequence of obstetric complications but a central driver of disease. Although inflammation plays a role in a broad range of obstetric complications, including spontaneous preterm labor and infection-associated preterm birth, the underlying biological pathways in these conditions are heterogeneous and often driven by distinct mechanisms such as microbial invasion of the amniotic cavity, decidual activation, or uterine inflammatory signaling independent of placental pathology. In contrast, preeclampsia and FGR represent prototypical models of placental-mediated disease, in which dysregulated immune responses at the maternal–fetal interface converge with abnormal placental vascular development [6,7]. These conditions share common features, including defective spiral artery remodeling, placental hypoxia, an imbalance in angiogenic factors, and systemic endothelial activation [8,9,10,11]. For this reason, they provide a particularly informative framework for understanding how inflammatory pathways contribute to placental dysfunction and maternal vascular disease.

The placenta is now recognized as a highly dynamic immunological organ in which trophoblasts, decidual stromal cells, and specialized immune populations—including uterine natural killer (uNK) cells, macrophages, and regulatory T cells—coordinate tolerance and defense at the maternal–fetal interface [3,12]. This equilibrium is sustained through multiple immunomodulatory pathways involving molecules such as HLA-G, programmed death ligand-1 (PD-L1), and Galectin-1, which dampen maternal cytotoxic responses and preserve fetomaternal tolerance [5,13,14,15]. However, environmental and pathological stressors- including placental hypoxia, oxidative stress, or sterile inflammatory signals- can disrupt this balance and reprogram the local immune environment toward pathological inflammation [5,14].

In preeclampsia and FGR, such immune dysregulation contributes to defective spiral remodeling, increased production of antiangiogenic factors such as soluble fms-like tyrosine kinase-1 (sFlt-1), and widespread endothelial dysfunction, hallmarks of placental insufficiency observed in both conditions [16,17,18,19,20]. Recent studies have identified activation of the NLRP3 inflammasome, an intracellular multiprotein complex involved in innate immune signaling, as a key mediator linking trophoblast stress to systemic maternal inflammation [15,21]. Activation of the NLRP3 pathway promotes maturation of the proinflammatory cytokines interleukin (IL)-1β and IL-18, which in turn drive Th1/Th17 polarization, disrupt uNK cell regulation, and exacerbate oxidative stress in placental tissues [22,23,24]. Additional cytokines such as tumor necrosis factor-α (TNF-α), IL-6, and IL-17 further amplify endothelial activation by increasing vascular permeability, reducing nitric oxide bioavailability, and promoting oxidative injury [25,26,27]. Emerging spatial and single-cell transcriptomic studies reveal how these inflammatory circuits are organized within the placenta, shedding light on cellular communication across the maternal–fetal interface [12,28].

Scientific advances have begun to translate into preventive and therapeutic strategies. Low-dose aspirin initiated early in gestation reduces the incidence of placental-mediated complications, likely through combined effects on platelet activation, angiogenic balance, and inflammatory signaling pathways [29]. Complementary approaches—including optimization of maternal nutrition and managing metabolic inflammation—are gaining support as regulators of immune homeostasis during pregnancy [30,31,32,33]. In parallel, emerging therapeutic strategies are being explored, including inflammasome inhibitors (e.g., MCC950), cytokine-targeted biologic therapies, and mesenchymal stem cell-based interventions [34]. Although these approaches remain largely experimental, they represent promising avenues for therapies aimed at restoring placental immune balance. However, their clinical translation demands thorough evaluation of maternal–fetal safety, dose optimization, and long-term developmental effects.

Despite substantial progress in placental immunology, critical gaps remain in understanding how immune dysregulation at the maternal–fetal interface translates into placental insufficiency and systemic endothelial dysfunction [15,16,28]. Preeclampsia and FGR are frequently considered separate clinical entities, yet accumulating evidence suggests that both emerge from defective spiral artery remodeling, placental ischemia, and the release of antiangiogenic and proinflammatory mediators—including sFlt-1, soluble endoglin, IL-6, and TNF-α—linking placental pathology to systemic maternal vascular dysfunction [8,10,17,18,35,36]. This review focuses primarily on preeclampsia and FGR as clinically relevant models of inflammation-driven placental disease. By synthesizing current evidence on inflammatory signaling at the maternal–fetal interface, we aim to integrate molecular mechanisms, biomarkers, and emerging therapeutic strategies to improve pregnancy outcomes and reduce global disparities in maternal and neonatal health.

## 2. Methodology

A narrative literature review was conducted to synthesize current knowledge on inflammatory mechanisms at the maternal–fetal interface and their contribution to placental dysfunction and pregnancy complications. The objective was to integrate experimental, translational, and clinical evidence regarding immune pathways involved in placental disease, with particular emphasis on fetal growth restriction and hypertensive placental disorders.

### 2.1. Literature Search Strategy

A structured search of the scientific literature was performed in PubMed/MEDLINE and Scopus databases. The search included studies published between January 1989 and November 2025, a timeframe selected to capture the development of modern concepts in placental immunology, angiogenic imbalance, and inflammatory signaling in pregnancy.

Search strategies combined Medical Subject Headings (MeSH) and free-text terms related to placental inflammation and immune regulation in pregnancy. The following key terms and their combinations were used:

- Preeclampsia;
- Fetal Growth Restriction;
- Placental Dysfunction;
- Inflammation;
- Inflammasome;
- Endothelium;
- Placenta;
- Immunology;
- Cytokines;
- Angiogenic imbalance;
- Maternal–fetal interface.

Boolean operators (AND, OR) were applied to generate search strings such as:

- (Preeclampsia OR Fetal Growth Restriction) AND (Inflammation OR Cytokines OR Immunology);
- Placenta AND (Inflammasome OR NLRP3);
- Placental dysfunction AND endothelial activation.

Reference lists of key publications and relevant review articles were also manually screened to identify additional studies not captured in database searches.

### 2.2. Eligibility Criteria

Articles were considered eligible if they addressed immune or inflammatory mechanisms related to placental biology or placental-mediated pregnancy complications. Eligible study designs included clinical studies, cohort studies, translational research, experimental models, and narrative and systematic reviews. Only articles published in English and available in full-text format were included. Studies focusing primarily on infection-driven preterm birth or unrelated obstetric conditions without a clear link to placental immune regulation were excluded unless they provided mechanistic insights relevant to placental inflammation.

### 2.3. Study Selection Process

The selection process was conducted in three stages. First, articles were initially screened by title to exclude studies that were clearly unrelated. Second, abstracts were reviewed to assess their relevance to the study objectives. Finally, full-text manuscripts were evaluated to confirm their contribution to the understanding of inflammatory mechanisms, placental dysfunction, or immune-mediated pathways in pregnancy. Priority was given to high-quality evidence, including well-designed cohort studies, translational research, and landmark experimental studies that have shaped current understanding of placental immunology.

## 3. Immune Development in the Placenta

**Figure 1:**
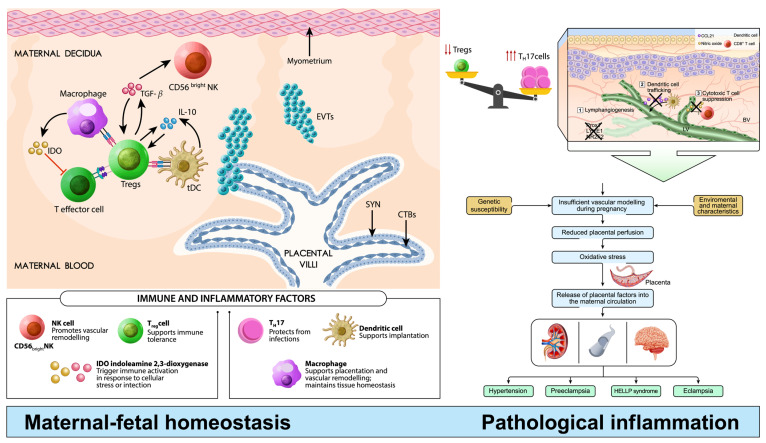
Immune homeostasis and dysregulation at the maternal–fetal interface in physiological pregnancy and preeclampsia. The left panel illustrates the state of maternal–fetal immune homeostasis required to maintain a healthy pregnancy. Within the decidua and placental villi, coordinated interactions between decidual NK cells, T cells (including regulatory T cells [Tregs] and effector T cells [Teff cells]), and macrophages support immune tolerance, appropriate trophoblast invasion, and physiological spiral artery remodeling, ensuring adequate placental perfusion. In this context, indoleamine 2,3-dioxygenase (IDO) activity contributes to immune regulation by catabolizing tryptophan, limiting Teff-cell proliferation and activation, and promoting a tolerogenic microenvironment. The right panel depicts an immune imbalance associated with placental dysfunction and preeclampsia. This state is characterized by reduced regulatory T cells (Tregs) and a shift toward proinflammatory T helper 17 (Th17) responses, accompanied by altered immune cell function. These changes impair implantation and spiral artery remodeling, leading to placental insufficiency. The resulting placental stress contributes to systemic maternal endothelial dysfunction and the clinical manifestations of preeclampsia.

The placenta plays a central role in pregnancy, mediating the delicate balance between maternal immune tolerance toward the fetus—a semi-allograft genetically distinct from the mother—and protection against infection [37,38]. The maternal–fetal interface, formed by the decidua and fetal trophoblast-derived tissues, constitutes a highly specialized immunological environment in which the maternal immune system adapts to sustain fetal growth while maintaining antimicrobial defense [39,40]. Successful pregnancy requires a tightly regulated immune equilibrium, characterized by coordinated interactions among decidual natural killer (NK) cells, T cells—including regulatory T cells (Tregs)—and macrophages, which promote tolerance, controlled trophoblast invasion, and appropriate spiral artery remodeling, ensuring adequate placental perfusion [37,38,41] (Figure 1).

Within this context, mechanisms such as indoleamine 2,3-dioxygenase (IDO) activity contribute to immune regulation by limiting effector T-cell proliferation and promoting a tolerogenic microenvironment. Disruption of this balance results in immune dysregulation, characterized by reduced Treg numbers, a shift toward proinflammatory T helper 17 (Th17) responses, and altered immune cell function. These changes impair implantation and vascular remodeling, leading to placental insufficiency and contributing to systemic maternal endothelial dysfunction, a hallmark of preeclampsia (Figure 1).

### 3.1. Immune Cells at the Maternal–Fetal Interface

Immune cell populations at this interface are recruited and regulated by cytokines secreted by trophoblasts and decidual stromal cells [42,43]. In early gestation, dNK cells account for approximately 70% of immune cells, macrophages for 20%, and Tregs for 10% [3,4,12,44]. Smaller populations include B lymphocytes, mast cells, and dendritic cells [3,4].

#### 3.1.1. Natural Killer Cells

dNK cells, located around invading extravillous trophoblasts, display a pregnancy-specific phenotype (CD56^bright^CD16^−^) shaped by interleukin-15 (IL-15) and transforming growth factor-β (TGF-β) [4,45,46]. They facilitate implantation, trophoblast invasion, and spiral artery remodeling by secreting IL-8, angiogenic mediators, and interferon-inducible protein-10 (IP-10) [45]. Their numbers decline during gestation, with Tregs assuming a more prominent immunoregulatory role [15,38,46]. Ultimately, this finely tuned balance of immune activation and tolerance at the maternal–fetal interface is fundamental to successful gestation.

#### 3.1.2. Decidual Macrophages

Macrophages in the decidua exhibit marked plasticity, responding dynamically to local microenvironmental cues [45]. They serve as primary antigen-presenting cells, recruited through trophoblast-derived IL-10 and macrophage colony-stimulating factor [47]. Two functional phenotypes predominate: pro-inflammatory M1 macrophages—most abundant during implantation and near term—and anti-inflammatory M2 macrophages, which dominate during established placentation [47]. Both subsets contribute to spiral artery remodeling, clearance of apoptotic cells, and antimicrobial defense by secreting vascular endothelial growth factor (VEGF) and matrix metalloproteinases (MMPs) [47,48]. Through this coordinated functional versatility, decidual macrophages play an essential role in sustaining tissue homeostasis and supporting a successful pregnancy.

#### 3.1.3. Regulatory T Cells

Tregs play a central role in maintaining immune tolerance during pregnancy, helping the mother’s immune system accept the semi-allogeneic fetus while still protecting against infection. Tregs secrete anti-inflammatory cytokines, including TGF-β and IL-10, promoting tolerance to fetal antigens, supporting implantation, and limiting excessive inflammation [49]. At the maternal–fetal interface, Tregs suppress excessive inflammation, promote vascular remodeling, and support healthy placental development by secreting cytokines such as IL-10 and TGF-β. Their population expands throughout gestation, and direct interactions between trophoblasts and Tregs appear critical for their differentiation and function [49,50]. When Treg number or function is reduced—or pro-inflammatory T-cell subsets dominate—immune imbalance can drive endothelial dysfunction, placental malperfusion, and tissue injury. This dysregulation has been implicated in complications including preeclampsia, fetal growth restriction, recurrent pregnancy loss, and preterm birth [37,38]. Although therapeutic strategies that boost Treg activity (e.g., targeted immunomodulation, metabolites influenced by the microbiome, or cell-based approaches) are under investigation, clinical translation remains early; restoring immune tolerance without compromising host defense remains the key challenge.

#### 3.1.4. Hofbauer Cells (HBCs)

HBCs are fetal-derived macrophages located within the villous stroma of the placenta and are present from the earliest stages of pregnancy (approximately day 18 post-conception), even before the establishment of the embryonic circulation. Transcriptomic studies indicate that these cells originate from primitive yolk sac hematopoiesis, positioning them as foundational components of the placental immunological microenvironment [51]. Under physiological conditions, HBCs express gene programs associated with angiogenesis, extracellular matrix remodeling, and placental homeostasis. They secrete mediators such as IL-8, osteopontin, and MMP-9, thereby contributing to the development of the villous tree and early placental vasculogenesis. In addition to their trophic functions, HBCs participate in fetal immune defense. These cells respond to Toll-like receptor signaling, exhibit microbicidal activity, and maintain immunological tolerance at the maternal–fetal interface.

A distinctive immunological feature of HBCs is their predominant expression of CD74 within placental tissues, which integrates signals related to antigen presentation and inflammatory regulation through macrophage migration inhibitory factor (MIF). Emerging evidence suggests that alterations in this pathway may contribute to placental disease. In preeclampsia, reduced CD74 expression has been associated with a shift toward a pro-inflammatory HBC phenotype, characterized by increased production of cytokines and chemokines, including TNF-α, CCL5, and monocyte chemotactic protein-1 (MCP-1). This inflammatory polarization may disrupt trophoblast-machophage communication and contribute to impaired spiral artery remodeling, endothelial dysfunction, and fetal growth restriction [52].

#### 3.1.5. Myeloid-Derived Suppressor Cells (MDSCs)

Myeloid-derived suppressor cells (MDSCs) have emerged as critical regulators of maternal–fetal immune tolerance during pregnancy. During healthy gestation, granulocytic MDSCs (G-MDSCs) accumulate in the maternal peripheral blood, cord blood, and placenta, where they suppress T cell activation through arginase-1 expression and reactive oxygen species production and polarize CD4+ T cells toward a protective Th2 phenotype [53,54,55]. Mouse studies have demonstrated the causal importance of this expansion: MDSC depletion during early gestation leads to implantation failure and increased uterine T cell infiltration, while adoptive transfer of G-MDSCs rescues pregnancy in abortion-prone models [56,57]. Progesterone drives G-MDSC differentiation and activation through STAT3 signaling, linking hormonal changes in pregnancy directly to immunosuppressive cell expansion [57]. Multiple reviews have proposed that MDSCs may serve as a unifying mechanism underlying the diverse immune adaptations required for successful pregnancy (Zhao et al., 2016; Pang et al., 2023; Zhang et al., 2022) [58,59,60].

When MDSC expansion fails, pregnancy complications ensue. In preeclampsia, [61]. showed that the pregnancy-associated increase in G-MDSCs—but not monocytic MDSCs—is markedly inhibited in both maternal and cord blood, accompanied by significantly reduced serum arginase-1 levels, while Treg cell frequencies remain unchanged between preeclamptic and healthy pregnancies. This suggests that G-MDSC deficiency is a more distinctive immunological feature of preeclampsia than Treg dysfunction. For fetal growth restriction (FGR) [62], reported that continuous activation of polymorphonuclear MDSCs (PMN-MDSCs) during pregnancy is closely associated with fetal growth, using the SR-E1 marker to track PMN-MDSC function in humans [63] further elucidated the mechanism, demonstrating that placental PMN-MDSCs secrete growth-promoting factors via an OLFM4/galectin-3/HIF-1α axis; myeloid-specific Olfm4 knockout in female mice produced FGR with decreased placental PMN-MDSCs, and OLFM4 expression was reduced in placental PMN-MDSCs from human FGR pregnancies. Taken together, these findings position MDSC dysfunction as a shared immunological feature across preeclampsia and fetal growth restriction and suggest that therapeutic strategies aimed at restoring MDSC numbers or function could represent a novel approach to managing these pregnancy complications [55,58,64].

### 3.2. Mechanisms of Immune Adaptation and Tolerance

Several molecular pathways sustain maternal tolerance while preserving host defense:

- Human Leukocyte Antigen (HLA): Extravillous trophoblasts express non-classical HLA molecules (HLA-C, HLA-E, HLA-G, HLA-F) that inhibit cytotoxic immune responses and facilitate placentation. HLA-G promotes NK-cell secretion of fetal growth factors [65].
- B7 Protein Family: High expression of costimulatory molecules such as B7-H1 on trophoblasts interacts with PD-1 receptors on maternal T cells, suppressing Th17 responses and enhancing Treg activity [66].
- TNF Superfamily Members: Placental Fas ligand (FasL) and TNF-related apoptosis-inducing ligand (TRAIL) induce apoptosis in activated maternal T cells [65], preventing immune-mediated rejection.
- Pattern Recognition Receptors: Toll-like receptors (TLRs) and RIG-I-like receptors on trophoblasts detect pathogen-associated molecular patterns, initiating controlled inflammatory cascades that preserve defense without disrupting tolerance [67].
- Maternal–Fetal IgG Transfer: Immunoglobulin G is actively transported across the placenta during the second and third trimesters, conferring passive immunity to the neonate and infant [68].
- Interferon-γ (IFN-γ): Produced by syncytiotrophoblast, IFN-γ plays a crucial role in antiviral protection and local immune signaling [69].
- Other Immunomodulators: Galectin-1, sex hormones such as progesterone and estrogen, and complement regulatory proteins (CD46, CD55, CD59) collectively maintain immune equilibrium and protect the fetus from complement-mediated injury [13].

These molecular pathways establish a tightly regulated immunological balance in which tolerance to fetal antigens is maintained while antimicrobial defense is preserved, supporting normal placental development and protecting the fetus from immune-mediated injury. Emerging evidence further suggests that the placental and maternal microbiomes contribute to this regulatory network, influencing immune maturation and susceptibility to inflammation-mediated pregnancy complications.

#### Galectin-Mediated Glyco-Immune Regulation at the Maternal–Fetal Interface

Galectins are β-galactoside-binding lectins that provide an additional layer of glycan-dependent immune regulation at the maternal–fetal interface. Among them, Galectin-1 (Gal-1) and Galectin-9 (Gal-9) play central roles in maintaining immune tolerance during pregnancy by integrating carbohydrate recognition with immune checkpoint signaling [13,70,71,72]. Gal-1 promotes maternal–fetal tolerance by selectively inducing apoptosis of activated T cells, expanding Tregs, and modulating natural killer (NK) cell function. It induces apoptosis of activated CD45RO^+^ T cells via carbohydrate-dependent interactions with glycosylated surface receptors, activating AP-1 signaling, downregulating Bcl-2, and triggering caspase pathways [5,73]. This mechanism preferentially targets activated effector T cells while sparing resting populations, thereby limiting maternal anti-fetal responses [5,74]. Gal-1 also promotes tolerogenic dendritic cells and enhances IL-10–dependent Treg expansion, while suppressing Th1/Th17 differentiation and IFN-γ production [13,74,75]. In Gal-1–deficient murine models, fetal loss increases significantly, whereas recombinant Gal-1 restores tolerance through Treg expansion [13].

At the decidual level, Gal-1 contributes to the establishment of the CD56^bright^CD16^−^ decidual NK phenotype, characterized by angiogenic and immunoregulatory functions rather than cytotoxicity [76]. Its expression is hormonally regulated through estrogen-responsive elements within the LGALS1 promoter and modulated by redox-dependent conformational changes, linking endocrine and immune pathways during placentation [76]. Gal-9 exerts complementary effects by interacting with TIM-3, an immune checkpoint receptor expressed on NK cells and T cells [77]. At the maternal–fetal interface, trophoblast-derived Gal-9 binds TIM-3 on decidual NK cells, suppressing cytotoxic responses toward trophoblast cells while preserving antimicrobial competence [13]. TIM-3 expression increases during early gestation and is associated with enhanced immune regulation and reduced fetal loss in experimental models [77].

In T cells, Gal-9–TIM-3 signaling promotes expansion of highly suppressive TIM-3^+^ Tregs, particularly in cooperation with IL-27 signaling. These cells exhibit strong proliferative and contribute to immune equilibrium within the decidua [14,16]. Gal-9 further restrains Th1 and Th17 responses and promotes Foxp3^+^ Treg differentiation [78]. Together, Gal-1 and Gal-9 integrate glycan recognition with immune checkpoint pathways, coordinating regulation of effector T cells, NK cells, and regulatory populations. Within the broader network of placental immunoregulation—including HLA-mediated inhibition, cytokine balance, and complementary control—these lectins help maintain tolerance to fetal antigens while preserving antimicrobial defense, supporting placental development and fetal protection during pregnancy.

## 4. The Maternal Microbiome: A Hidden Regulator of Pregnancy

Throughout pregnancy, the maternal microbiome undergoes profound physiological shifts driven by hormonal, metabolic, and immunological changes. These alterations influence maternal health and fetal development, shaping susceptibility to conditions such as gestational diabetes mellitus and other noncommunicable diseases [79,80,81,82]. In preeclampsia, studies have demonstrated consistent alterations in gut microbiota composition, including increased abundance of *Proteobacteria* and *Actinobacteria*, accompanied by reduced levels of *Prevotella*, *Varibaculum*, *Lactobacillus*, and *Porphyromonas* [83]. These changes reflect a shift toward a proinflammatory microbial profile, supporting the hypothesis that maternal gut dysbiosis may contribute to systemic inflammation, endothelial dysfunction, and ultimately placental disease. Together, these findings underscore the maternal microbiome as a dynamic regulator of immune and metabolic homeostasis during pregnancy. Understanding how microbial dysbiosis contributes to disorders such as preeclampsia offers a promising path to early risk stratification and targeted interventions to improve maternal and neonatal outcomes.

## 5. Evidence of Altered Inflammation in Preeclampsia

**Figure 2:**
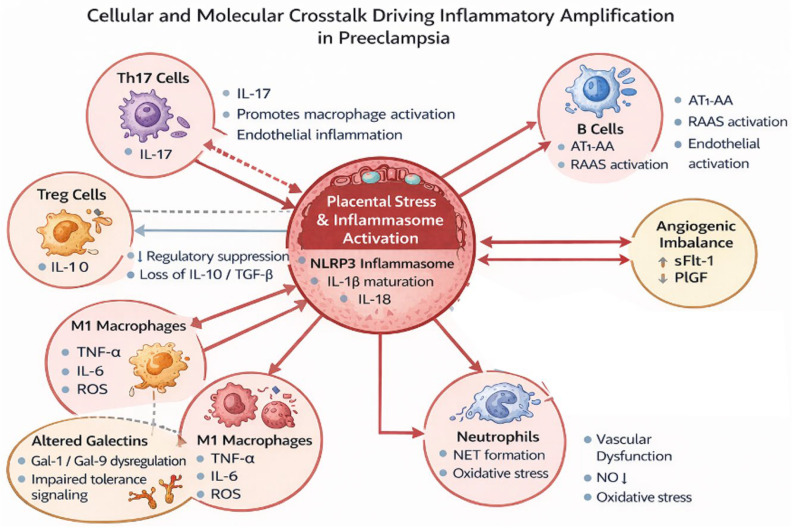
Cellular and molecular interactions driving inflammatory amplification in preeclampsia. Activation of the NLRP3 inflammasome within the placental microenvironment promotes a proinflammatory cascade through dynamic crosstalk among Th17 lymphocytes, classically activated (M1) macrophages, neutrophils, and B cells. This network enhances the production of key inflammatory mediators—including IL-1β, IL-6, TNF-α, and IL-17—which collectively amplify local and systemic immune activation. Concurrent reduction in regulatory T-cell (Treg) function, together with dysregulated galectin-mediated signaling, disrupts mechanisms of immune tolerance at the maternal–fetal interface. These alterations establish a self-reinforcing inflammatory circuit that contributes to placental dysfunction, endothelial activation, and the pathophysiological cascade characteristic of preeclampsia.

Normal pregnancy is characterized by a controlled, low-grade systemic inflammatory state that is essential for implantation, placental development, and preparation for parturition [84,85]. Compared with the non-pregnant state, pregnancy is associated with leukocytosis [84], increased complement activation, and dynamic shifts in peripheral leukocyte populations [84]. Distinct immunological phases have been described, reflecting changes in both innate and adaptive immunity [4,42,84,86]. This physiological inflammatory environment represents a balance between proinflammatory mediators—such as TNF-α, IL-2, IL-4, IL-6, IL-8, IL-10, interferon-γ (IFN-γ), and monocyte chemotactic protein-1 (MCP-1) [4,41,42]—and anti-inflammatory/regulatory mechanisms (Figure 2).

In preeclampsia, this finely regulated inflammatory equilibrium is destabilized, leading to a coordinated network of immune cell interactions that amplify proinflammatory signaling. Rather than isolated cytokine elevation, the disorder is characterized by reciprocal activation among Th17 cells, M1 macrophages, neutrophils, B cells, and reduced regulatory T-cell suppression. Central to this process is activation of the NLRP3 inflammasome, which promotes maturation of IL-1β and IL-18 and reinforces inflammatory crosstalk across immune subsets (Figure 2).

Preeclampsia shares some features with the inflammatory state of normal pregnancy, but the intensity of the response is markedly increased [8,86,87]. This exaggerated inflammation is thought to originate from syncytiotrophoblast stress due to hypoxia, oxidative stress, or both [5,84,85,87], leading to the release of proinflammatory cytokines and antiangiogenic factors [5,10,84]. The inflammatory response may be triggered by both exogenous danger signals (e.g., pathogens) and endogenous signals (products of trauma, ischemia, necrosis, or oxidative stress) [88,89,90]. It involves inflammatory leukocytes (granulocytes, macrophages, and NK cells) in concert with endothelial cells, platelets, and soluble mediators, such as complement [38,86].

Preeclampsia is conceptualized as a two-stage disorder [8,87]:

1. Pre-clinical Stage—Abnormal placentation and inadequate spiral artery remodeling, leading to placental hypoperfusion [14,91].
2. Clinical Stage—Systemic maternal endothelial dysfunction and hypertension [18,84,92].

Placental-derived antiangiogenic factors—such as increased sFlt-1 and sEng and decreased placental growth factor (PlGF)—are distinctive features of preeclampsia and contribute to widespread endothelial injury [20,92]. In summary, preeclampsia represents the convergence of dysregulated inflammation, imbalanced angiogenic signaling, and heightened long-term cardiovascular vulnerability [20,85,86]. Excess maternal inflammatory activation injures the endothelium and amplifies oxidative stress, whereas anti-angiogenic factors, such as sFlt-1 and sEng, disrupt placental perfusion and propagate systemic vascular dysfunction. These acute pathophysiologic pathways not only drive the clinical syndrome of hypertension, proteinuria, and multi-organ involvement but also leave a persistent imprint on maternal cardiovascular health, explaining the markedly increased risk of later hypertension, ischemic heart disease, and stroke [16,23,93]. Understanding how immune, vascular, and metabolic networks intersect in preeclampsia is essential for developing therapies that protect both immediate pregnancy outcomes and lifelong cardiovascular health.

### 5.1. Autophagy and Placental Stress Responses in Preeclampsia

Autophagy is a highly conserved intracellular degradation pathway that maintains cellular homeostasis by recycling damaged organelles, protein aggregates, and misfolded proteins [78,94]. In the placenta, basal autophagic activity plays an essential role in trophoblast differentiation, adaptation to hypoxic stress, and the regulation of oxidative balance [66,95]. Under physiological conditions, autophagy supports syncytiotrophoblast renewal and contributes to metabolic equilibrium within the developing placenta [95,96].

In preeclampsia, however, autophagic regulation appears to become dysregulated. Placental hypoxia and oxidative stress—hallmarks of impaired spiral artery remodeling—can trigger excessive or maladaptive autophagic responses [97,98]. Altered expression of key autophagy-related proteins, including LC3-II, Beclin-1, and p62, has been documented in preeclamptic placentas, suggesting disruption of normal autophagic flux [98,99]. Such alterations may promote trophoblast apoptosis, impair trophoblast invasion, and exacerbate placental insufficiency [97,98].

Autophagy also interacts closely with inflammatory signaling pathways. Experimental evidence indicates significant crosstalk between autophagic mechanisms and NLRP3 inflammasome activation. Impaired autophagy may lead to the accumulation of damaged mitochondria and increased production of reactive oxygen species, thereby facilitating inflammasome activation and enhanced IL-1β secretion [8,9,10]. Conversely, intact autophagic activity can limit excessive inflammatory signaling by promoting mitochondrial quality control and reducing oxidative stress, thereby protecting endothelial integrity [100,101].

Together, these findings position autophagy as a key regulatory pathway linking placental hypoxic stress, inflammasome activation, and trophoblast dysfunction. A deeper understanding of autophagic regulation in the placenta may therefore reveal novel therapeutic targets to restore cellular homeostasis and prevent inflammation-driven vascular injury in preeclampsia. Importantly, these stress-response pathways also intersect with adaptive immune dysregulation, further amplifying cytokine imbalance and contributing to systemic endothelial dysfunction.

### 5.2. Immune Dysregulation and Cytokine Imbalance

Since Medawar’s 1953 hypothesis that the fetus is an “allograft” [40,102], early theories posited that maternal immune privilege at the maternal–fetal interface was necessary to maintain pregnancy [65,103]. These models emphasized maternal immune suppression, reduced expression of major histocompatibility complex (MHC) antigens in fetal tissue, and a shift from Th1 to Th2 cytokine dominance [67,90,102,104]. Contemporary evidence, however, indicates that a balanced interplay between pro- and anti-inflammatory responses is essential for successful pregnancy [90,102]. In early gestation, implantation and placental development are inherently proinflammatory but are tightly regulated by anti-inflammatory mediators, such as IL-10 and Tregs [1,50].

In preeclampsia, this balance is disrupted [86,105]. There is an overactivation of Th1 and Th17 responses, reduced Treg activity, and increased activation of cytotoxic NK cells and autoreactive B cells [86,104]. This leads to increased innate immune activation in both the maternal circulation and the uteroplacental unit [84,87,90]. The result is superficial trophoblast invasion, poor spiral artery remodeling, and worsening placental ischemia with escalating oxidative stress [15,104,106,107].

### 5.3. Cytokines and Endothelial Dysfunction in Preeclampsia

Chronic immune activation in preeclampsia is characterized by elevated levels of proinflammatory cytokines, particularly TNF-α, IL-6, and IL-17 [25,26,86,104,108]. These act locally and systemically, perpetuating oxidative stress, vascular inflammation, and endothelial damage.

- TNF-α and IL-6: Promote endothelial adhesion molecule expression, increase vascular permeability, and impair endothelial nitric oxide synthase (eNOS) activity, reducing nitric oxide (NO) bioavailability and vasodilation [108,109]. Experimental models show that a two-fold increase in TNF-α elevates mean arterial pressure (MAP) and reduces renal plasma flow and glomerular filtration rate (GFR) [109,110]. IL-6 exerts similar effects and has been linked to increased plasma renin activity [109,111].
- IL-17: In animal models, IL-17 increases MAP, induces placental oxidative stress, and stimulates B cells to produce angiotensin II type 1 receptor autoantibodies (AT1-AA), exacerbating hypertension and vascular injury [104].

**Figure 3:**
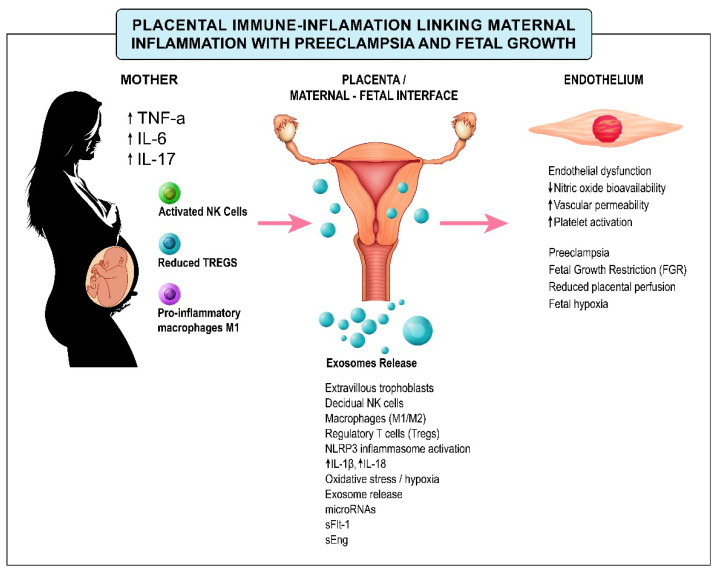
Maternal–placental inflammatory crosstalk linking systemic immune activation to endothelial dysfunction in preeclampsia and fetal growth restriction. This schematic illustrates the directional interplay between maternal systemic inflammation, placental immune signaling, and endothelial perturbation. Elevated maternal cytokines (e.g., TNF-α, IL-6, IL-17), activation of innate immune cells, and reduced regulatory T-cell activity influence the decidual and trophoblastic environment. Placental stress promotes inflammasome activation, oxidative imbalance, and release of bioactive mediators—including exosomes, microRNAs, and antiangiogenic factors—that propagate inflammatory and vascular signaling. The convergence of these processes contributes to impaired uteroplacental perfusion and endothelial dysfunction, linking maternal immune dysregulation with adverse pregnancy outcomes.

Together, these inflammatory and immunological disturbances integrate placental dysfunction with maternal endothelial injury, illustrating how maternal immune activation at the systemic level converges on placental signaling and endothelial dysfunction (Figure 3).

## 6. Inflammation and Fetal Growth Restriction

**Figure 4:**
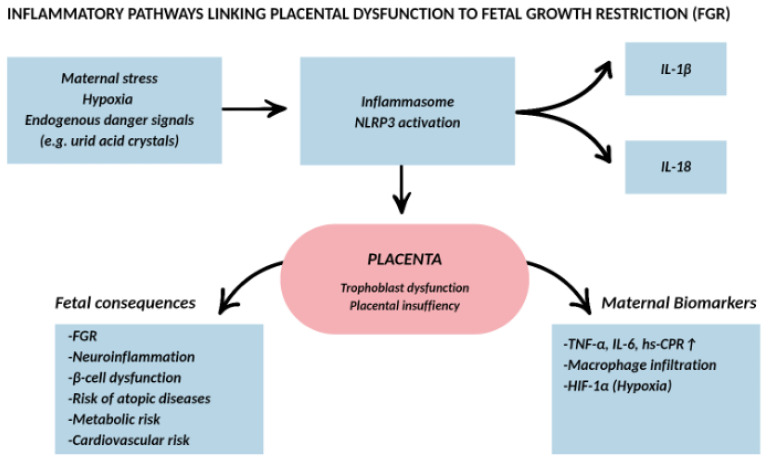
NLRP3 inflammasome–mediated inflammatory pathways contributing to fetal growth restriction. Maternal hypoxia and endogenous danger signals activate the placental NLRP3 inflammasome, promoting IL-1β and IL-18 maturation and trophoblast dysfunction. Sustained inflammasome signaling contributes to placental insufficiency and adverse fetal consequences, including growth restriction, neuroinflammatory changes, and long-term metabolic vulnerability. The diagram emphasizes inflammasome-driven mechanisms specific to FGR pathophysiology.

FGR is a complex clinical condition associated with increased perinatal morbidity and mortality [112,113]. It is primarily a consequence of placental insufficiency and alterations in the intrauterine inflammatory environment, both of which can have lasting effects on perinatal and long-term health [114,115,116]. FGR is defined as the failure of a fetus to achieve its genetically determined growth potential [113]. Clinically, the most widely accepted criterion is an estimated fetal weight below the 10th percentile for gestational age, as defined by standardized growth charts [117]. Multiple etiological factors contribute to FGR, with inflammation playing a pivotal role in its pathogenesis [118]. Excessive placental inflammation can be triggered by maternal stress, hypoxia, and endogenous danger signals such as uric acid crystals [6,22,88]. These stimuli activate placental inflammasomes—particularly NLRP3—promoting IL-1β–dependent signaling that impairs trophoblast function and contributes to placental insufficiency. Although inflammasome activation is also implicated in preeclampsia, its sustained activation appears particularly relevant in the context of placental insufficiency–driven fetal growth restriction (Figure 4).

The NLRP3 inflammasome, a multiprotein complex, mediates the maturation of proinflammatory cytokines such as IL-1β and IL-18, which are critical in amplifying inflammatory responses [22,26]. Experimental models demonstrate that sustained maternal inflammation can disrupt fetal metabolic programming, affecting skeletal muscle glucose metabolism and β-cell function, with potential implications for future metabolic disease risk [119]. Placental inflammation is also associated with increased macrophage infiltration and a proinflammatory cytokine profile in both placental tissue and maternal serum [6,105,120]. Furthermore, inflammation in FGR may extend beyond the placenta, affecting other systems such as the central nervous system—where neuroinflammation can impair brain development [121,122,123]—and the skin, where inflammatory changes may predispose to childhood atopic diseases [81].

Assessment of inflammatory involvement in FGR includes evaluation of both systemic and local biomarkers. Elevated maternal serum levels of TNF-α, IL-6, and high-sensitivity C-reactive protein (hs-CRP) have been reported in pregnancies complicated by FGR, with correlations to adverse neonatal outcomes such as low birth weight and reduced Apgar scores [88,119,124]. Histological and immunohistochemical analyses of placental tissue reveal increased macrophage density and a predominance of proinflammatory markers [125,126]. Additionally, hypoxia-induced factors such as hypoxia-inducible factor 1-alpha (HIF-1α) accumulate in placentas of pregnancies complicated with FGR, linking placental hypoxia to sterile inflammation and dysfunction [127]. The inflammatory milieu associated with FGR may have lasting effects on both the fetus and the mother. Children with a history of poor postnatal growth demonstrate elevated circulating inflammatory markers—such as C-reactive protein, hepatocyte growth factor, IL-8, and TNF-α—compared with age-matched controls [128]. This low-grade inflammation may increase the risk of future metabolic and cardiovascular disease. Animal studies indicate that FGR can blunt the neonatal proinflammatory response to infectious stimuli, mediated by reduced nuclear factor kappa B (NF-κB) activation [119]. This suggests impaired innate immunity and increased susceptibility to infection. On the maternal side, pregnancies complicated by FGR often show elevated proinflammatory cytokines, reinforcing the concept of a shared maternal–fetal inflammatory environment [6]. Collectively, these findings position placental inflammation not only as a determinant of fetal growth restriction but as part of a broader spectrum of immune-mediated placental insufficiency with shared maternal vascular consequences.

## 7. Immune Dysregulation in Placental Insufficiency: Clinical Implications and Translational Perspectives

**Figure 5:**
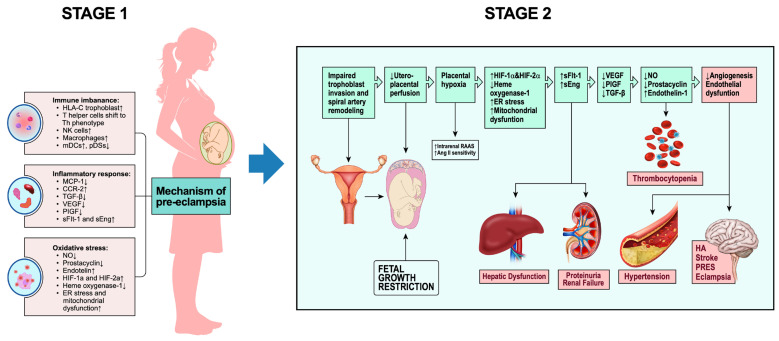
Inflammatory and Angiogenic Cascade Leading to Endothelial Dysfunction and Clinical Manifestations in preeclampsia and fetal growth restriction. This schematic illustrates the progressive transition from regulated physiological inflammation to a dysregulated state characterized by sustained immune activation and angiogenic imbalance. Excessive production of proinflammatory cytokines and antiangiogenic mediators promotes endothelial dysfunction, oxidative stress, and impaired uteroplacental perfusion. As vascular injury intensifies, maternal and fetal consequences emerge along a continuum, culminating in the clinical phenotypes of preeclampsia and fetal growth restriction, which may present independently or concurrently.

From a clinical perspective, both preeclampsia and fetal growth restriction are consistently associated with elevated circulating inflammatory mediators, including key Th1/Th17-associated cytokines such as TNF-α, IL-6, and IL-17 [27,86,90,104]. These cytokines amplify oxidative stress in placental tissue, aggravating endothelial dysfunction and contributing to the most acute and clinically severe stages of the disease. However, interpreting these biomarkers in clinical practice remains challenging, as inflammation can also be influenced by maternal characteristics such as advanced age, prolonged interpregnancy interval, obesity, and comorbidities, including chronic hypertension, diabetes, renal disease, and autoimmune conditions (Figure 5).

Dysregulation of galectin-mediated glyco-immune signaling further contributes to this cascade, as reduced Gal-1 and altered Gal-9 activity impair immune tolerance and vascular remodeling, reinforcing the transition from physiological adaptation to pathological inflammation.

Beyond heightened inflammation, preeclampsia is also characterized by impaired anti-inflammatory regulation [86]. A consistent finding across studies is the reduction in Tregs and decreased IL-10 production—both essential for maintaining immunological tolerance at the maternal–fetal interface. Several reports indicate that Treg cell depletion is more pronounced in early-onset, severe preeclampsia compared with late-onset or milder forms [91,102,129]. Reduced Treg function promotes increased trophoblast apoptosis, shallow trophoblast invasion, and inadequate spiral artery remodeling [49,102], highlighting a mechanistic link between immune dysregulation and the placental malperfusion that precedes clinical disease [86].

IL-10 plays a central role in this process. As a key anti-inflammatory cytokine that restrains Th1-driven inflammation and supports Treg differentiation, its reduction in preeclampsia further skews the maternal immune milieu toward a cytotoxic, proinflammatory state. This imbalance contributes to hypertension, endothelial activation, and ultimately impaired fetal growth [5,84]. Together, these findings underscore the importance of immune homeostasis for optimal pregnancy outcomes. Clinically, they highlight potential avenues for intervention: therapies that enhance Treg function, restore IL-10 signaling, or attenuate excessive cytokine activity could help rebalance inflammatory pathways and improve maternal and perinatal outcomes. Although still experimental, strategies such as Treg cell transfer, IL-10–based therapies, and selective immunomodulators hold promise and warrant further investigation in well-designed translational studies [86]. Strengthening the bridge between immunopathology and clinical care is essential. By identifying immune signatures that distinguish pathological inflammation from physiological adaptation, future strategies may enable earlier detection, refined risk stratification, and mechanism-oriented interventions aimed not only at preventing acute obstetric complications but also at mitigating long-term maternal cardiovascular risk.

## 8. Therapy Development

Therapeutic strategies for pregnancy-associated inflammatory disorders must achieve a delicate balance between modulating the maternal immune response and ensuring fetal safety. The primary objective is to attenuate excessive inflammation—implicated in adverse outcomes such as preeclampsia, FGR, and preterm birth—while preserving adequate host defense against infections. Current management combines evidence-based interventions from established clinical guidelines with emerging approaches that target specific inflammatory pathways (Table 1). Understanding how these interventions modulate the maternal–fetal immune landscape is essential for developing more precise and effective therapies.

### 8.1. Established Therapies

#### 8.1.1. Low-Dose Aspirin

Low-dose aspirin (LDA), initiated before 16 weeks of gestation, reduces the incidence of preeclampsia and other placental-mediated complications. Its mechanism involves inhibition of platelet aggregation and modulation of inflammatory responses via selective cyclooxygenase-1 (COX-1) blockade [29,147]. Emerging clinical guidelines increasingly support the use of LDA in women identified as high risk for preeclampsia following first-trimester screening. Evidence from randomized trials and meta-analyses demonstrates that initiating LDA—ideally before 16 weeks—significantly reduces the risk of preeclampsia, particularly early-onset disease, and associated outcomes, including preterm birth and fetal growth restriction [29,147]. Screening-based strategies enable targeted treatment of women most likely to benefit, thereby optimizing the risk–benefit balance. Collectively, these data underpin current recommendations endorsing prophylactic LDA as a cornerstone of prevention in appropriately selected pregnancies.

#### 8.1.2. Anticoagulants

Low molecular weight heparin (LMWH) is indicated in selected high-risk pregnancies, particularly in the context of thrombophilia or recurrent pregnancy loss. Beyond its anticoagulant properties, LMWH exhibits anti-inflammatory effects by modulating complement activation and leukocyte trafficking [93]. A growing body of evidence suggests that adjunctive LMWH may confer benefit in women at high risk of preeclampsia who do not have thrombophilia. In a meta-analysis of 10 randomized trials (1758 participants), LMWH significantly reduced the incidence of preeclampsia, with risk reduction confined mainly to studies in which low-dose aspirin was the primary intervention. The combination of LMWH and LDA was also associated with fewer preterm births and fewer cases of fetal growth restriction, without affecting placental abruption rates. Overall, these findings support the selective use of LMWH as an adjunct to LDA in carefully defined high-risk populations [148].

#### 8.1.3. Corticosteroids

Antenatal corticosteroids remain a cornerstone when preterm delivery is anticipated in pregnancies complicated by fetal growth restriction or preeclampsia, primarily because they accelerate fetal lung maturation and reduce neonatal respiratory distress, intraventricular hemorrhage, and death [149,150]. Although they do not directly treat placental insufficiency or maternal disease, their use can safely extend the window for optimizing delivery timing and adjunctive care. In these high-risk contexts, clinicians must balance benefits against potential maternal effects—including transient hyperglycemia, fluid retention, and a small increase in infectious risk—particularly in women with diabetes or severe hypertension. Overall, a single standard course before early preterm birth is strongly supported, while repeat dosing requires individualized consideration [149,150].

These established therapies delineate the current therapeutic landscape—effective in specific contexts yet fundamentally constrained by the need to safeguard the maternal–fetal unit. This limitation underscores an urgent scientific priority: to design next-generation immunomodulatory approaches that selectively suppress pathological inflammation while preserving fetal development. As insights into placental immunobiology expand, the development of truly targeted, precision therapies in pregnancy is shifting from possibility to near inevitability.

### 8.2. Adjunctive and Supportive Interventions

#### 8.2.1. Antioxidants and Nutritional Modulation

Oxidative stress is a key contributor to placental inflammation [151,152]. Although antioxidants such as vitamins C and E have been extensively evaluated, large clinical trials have not demonstrated consistent benefit in preventing preeclampsia [90]. Melatonin, with potent antioxidant and vasoprotective properties, has shown promising results in small-scale studies [153,154], suggesting a potential role for targeted antioxidant therapy. Experimental data demonstrate that melatonin reduces placental oxidative stress and enhances antioxidant defenses without altering key anti-angiogenic mediators, and that it partially protects endothelial function in vitro. In a phase I clinical study involving women with established preeclampsia, melatonin administration was safe for both mother and fetus [153]. It was associated with a meaningful prolongation of pregnancy, along with reduced requirements for escalation of antihypertensive therapy [153]. Although significant biochemical markers of disease severity remained unchanged, these findings suggest that melatonin may attenuate maternal endothelial injury and help stabilize the disease long enough to improve perinatal outcomes, warranting larger confirmatory trials.

Recent high-quality evidence challenges earlier assumptions regarding routine calcium supplementation for the prevention of preeclampsia [155]. Across large, prospectively registered randomized trials, calcium showed little to no effect on preeclampsia, preterm birth, or major maternal and perinatal outcomes, regardless of baseline intake or dose. Overall, current data suggest that dietary calcium alone is unlikely to modify preeclampsia risk meaningfully and should not replace proven preventive strategies in high-risk pregnancies [155].

#### 8.2.2. Lifestyle and Microbiome Modulation

Optimizing maternal diet, engaging in regular moderate physical activity, and using probiotic supplementation are under investigation as strategies to modulate systemic inflammation and enhance immune tolerance during pregnancy [30,156]. Lifestyle and microbiome modulation are emerging areas of interest for reducing the risk of preeclampsia, though the evidence remains preliminary. Observational and mechanistic studies suggest that healthy dietary patterns, appropriate weight gain, physical activity, and smoking avoidance may attenuate systemic inflammation, improve metabolic function, and support vascular health—pathways central to preeclampsia [157]. Parallel research indicates that alterations in the gut, vaginal, and placental microbiomes may influence immune tolerance, endothelial function, and oxidative stress, suggesting that probiotics, prebiotics, or diet-driven microbial shifts could play a preventive role. However, high-quality randomized trials are scarce, and standardized interventions have not yet been defined. For now, lifestyle optimization should be encouraged as part of comprehensive antenatal care, while microbiome-targeted therapies remain promising but investigational.

### 8.3. Emerging and Experimental Therapies

#### 8.3.1. Cytokine-Targeted Biologics

Monoclonal antibodies that neutralize pro-inflammatory cytokines (e.g., anti–TNF-α, anti–IL-6) have been successful in treating autoimmune diseases and are now under consideration for the treatment of pregnancy-related inflammatory disorders [158]. Their capacity to exert highly specific immunomodulatory effects makes them an appealing, although still experimental, therapeutic avenue. Experimental models suggest these cytokines contribute to endothelial dysfunction, placental malperfusion, and systemic oxidative stress, and targeted blockade can partially restore vascular homeostasis. Clinically, experience in pregnant patients largely comes from women treated for autoimmune diseases, where selected agents (e.g., certain anti–TNF drugs) have shown acceptable safety profiles when carefully monitored; however, robust randomized data specific to hypertensive or placental disorders are lacking. At present, cytokine-neutralizing antibodies remain investigational for pregnancy-specific indications, and their future role will depend on trials that balance maternal benefit with fetal immune and developmental safety.

#### 8.3.2. Inflammasome Inhibitors

Given the pivotal role of NLRP3 inflammasome activation in placental inflammation and FGR, small-molecule inhibitors such as MCC950 are being tested in preclinical models [159]. By targeting upstream inflammatory signaling, these agents may help mitigate placental injury. Inflammasome inhibitors, particularly those targeting NLRP3, offer a novel approach for pregnancy disorders in which excessive IL-1β and IL-18 signaling drives endothelial and placental injury, such as preeclampsia and fetal growth restriction [160]. Preclinical models suggest that pharmacologic blockade of inflammasome activation can attenuate oxidative stress, vascular dysfunction, and placental inflammation, thereby improving maternal hemodynamics and fetal growth [160]. However, clinical experience in pregnancy is virtually absent, and concerns remain regarding host defense and fetal immune development. At present, inflammasome inhibition is a promising but purely experimental strategy that requires rigorous safety and efficacy trials before translation to obstetric care.

#### 8.3.3. Cell-Based Therapies

Mesenchymal stem cell (MSC) therapy represents a novel approach to promote immune tolerance and facilitate tissue repair at the maternal–fetal interface [161]. Early data indicate that MSCs possess strong immunomodulatory and regenerative potential, positioning them as a promising frontier in perinatal therapeutics. Cell-based therapies are emerging as an innovative strategy for preeclampsia, aiming to repair rather than merely temporize placental and endothelial injury [161]. Mesenchymal stromal cells, endothelial progenitor cells, and regulatory T-cell–based approaches have been shown in preclinical models to improve uteroplacental perfusion, dampen inflammation, and restore endothelial function, primarily through paracrine and immunomodulatory mechanisms. Early-phase studies outside pregnancy suggest acceptable safety profiles for some platforms, but robust clinical data in pregnant populations are lacking. For now, cell-based therapies remain experimental, offering a compelling future avenue for disease modification in severe or early-onset preeclampsia [161].

#### 8.3.4. Safety Considerations

The administration of anti-inflammatory agents during pregnancy requires rigorous assessment of their teratogenic potential, effects on fetal immune development, and potential long-term effects on offspring. Current guidelines advocate individualized risk–benefit evaluation and preferentially use therapies with well-established safety profiles [162,163]. While current and emerging therapeutic approaches offer promising avenues for managing pregnancy-associated inflammatory disorders, ongoing research is advancing toward even more targeted and biologically precise interventions. Among these, exosome-based strategies are among the most innovative and rapidly evolving frontiers.

## 9. Future Perspectives: Emerging Role of Exosomal Signaling

Growing evidence indicates that placental-derived exosomes participate in immune and vascular communication at the maternal–fetal interface. Exosomes are nanoscale extracellular vesicles that transport bioactive cargo, including proteins, mRNA, and microRNAs, enabling coordinated signaling between placental and maternal tissues and influencing inflammatory and angiogenic pathways [164,165,166,167]. During physiological pregnancy, trophoblast-derived exosomes contribute to immune modulation and the maintenance of maternal–fetal tolerance, facilitating the transfer of regulatory molecules [166,168,169,170,171].

In preeclampsia and FGR, alterations in exosomal composition have been reported, including enrichment of proinflammatory mediators and antiangiogenic factors such as sFlt-1 [172,173]. These observations suggest that exosomes may act as intermediaries linking placental stress to systemic inflammatory signaling and endothelial dysfunction, although the precise mechanisms remain incompletely defined.

Experimental studies using mesenchymal stromal cell-derived extracellular vesicles have demonstrated immunomodulatory effects and partial restoration of placental vascular remodeling in preclinical models [146]. While these observations highlight the therapeutic potential of exosome-based strategies, further mechanistic studies are needed before they can be translated into clinical practice. Collectively, exosomal signaling represents a promising but still evolving area of investigation within the broader landscape of immune-mediated placental disease, particularly in the context of preeclampsia and FGR.

## 10. Conclusions

Inflammation is an intrinsic component of normal pregnancy, orchestrating implantation, placental development, and the physiological onset of labor. However, when inflammatory regulation becomes disrupted at the maternal–fetal interface, these protective immune mechanisms may shift toward pathological signaling that contributes to placental and vascular disease. The evidence reviewed here supports the concept that preeclampsia and fetal growth restriction represent interconnected manifestations of inflammation-driven placental dysfunction, arising from disturbances in the delicate equilibrium between maternal immune tolerance and vascular adaptation.

Within this inflammatory landscape, immune cell imbalance, inflammasome activation, cytokine amplification, and angiogenic disruption converge to impair trophoblast invasion, compromise spiral artery remodeling, and reduce placental perfusion. These local placental alterations propagate systemic effects, ultimately contributing to maternal endothelial dysfunction and adverse fetal outcomes. Viewing these disorders through a unified immunological framework helps bridge molecular mechanisms to clinical expression and reinforces the concept that placental disease arises from complex interactions among immune signaling, vascular biology, and environmental stressors. Importantly, this perspective highlights opportunities for precision approaches targeting specific inflammatory and angiogenic pathways, while preserving the physiological immune adaptations required for successful gestation.

Advances in molecular profiling, systems immunology, and translational research will be essential to further characterize the inflammatory networks that shape placental function. Such efforts may facilitate the development of targeted preventive and therapeutic strategies, ultimately improve maternal and neonatal outcomes and reduce global disparities in pregnancy-related complications.

## Figures and Tables

**Table 1:** Comparative Therapies aimed to modulate inflammation during pregnancy.

Category	Type	Mechanism of Action	Evidence Level
Low-Dose Aspirin [29,130,131,132]	Established	COX-1 inhibition → Reduction of platelet aggregation & inflammation	High—Multiple RCTs in PE prevention
Low Molecular Weight Heparin [133,134,135,136]	Established	Anticoagulation + modulation of complement & leukocyte trafficking	Moderate—Observational studies & some RCTs
Antenatal Corticosteroids [137,138]	Established	Promote fetal lung maturation, transient anti-inflammatory effect	High—Standard of care in threatened preterm birth
Antioxidants & Nutritional Modulation [139,140]	Adjunctive	Reduce oxidative stress, improve endothelial function	Low to Moderate—Inconsistent RCT results
Lifestyle & Microbiome Modulation [141]	Adjunctive	Improve immune tolerance & reduce systemic inflammation	Low—Ongoing trials
Cytokine-Targeted Biologics [142,143]	Emerging	Neutralize pro-inflammatory cytokines (e.g., TNF-α, IL-6)	Experimental—Limited pregnancy-specific data
Inflammasome Inhibitors [144]	Emerging	Block NLRP3 inflammasome activation	Preclinical—Animal models
Cell-Based Therapies [145,146]	Emerging	Promote immune tolerance & tissue repair at maternal–fetal interface	Preclinical—Early-phase studies

## Data Availability

The original contributions presented in this study are included in the article. Further inquiries can be directed to the corresponding author.

## Abbreviations

FGR	Fetal growth restriction
Th1	T-helper 1 cells
Th2	T-helper 2 cells
HDP	Hypertensive disorders of pregnancy
uNK	Uterine natural killer cells
HLA-G	Human Leukocyte Antigen-G
PD-L1	Programmed Death-Ligand 1
sFlt-1	soluble fms-like tyrosine kinase-1
NLRP3	NOD-like receptor family, pyrin domain containing 3
IL	Interleukin
TNF-α	Tumor necrosis factor-α
dNK	Decidual natural killer cells
CD56	Cluster of Differentiation 56
IP-10	Interferon-inducible protein-10
VEGF	Vascular endothelial growth factor
Tregs	Regulatory T cells
TGF-β	Transforming Growth Factor-Beta
FasL	Placental Fas ligand
TLRs	Toll-like receptors
PTB	Preterm birth
LBW	Low birth weight
MCP-1	Monocyte chemotactic protein-1
PlGF	Placental growth factor
sEng	Soluble endoglin
MHC	Histocompatibility complex
eNOS	Endothelial nitric oxide synthase
NO	Nitric oxide
MAP	Mean arterial pressure
HIF-1α	Hypoxia-inducible factor 1-alpha
NF-κB	Nuclear factor kappa B
LDA	Low-dose aspirin
COX-1	Cyclooxygenase-1
LMWH	Low molecular weight heparin
MSC	Mesenchymal stem cell
miRNAs	microRNAs
HBCs	Hofbauer cells
